# Hydrogenolysis of Glycerol to Propylene Glycol: Energy, Tech-Economic, and Environmental Studies

**DOI:** 10.3389/fchem.2021.778579

**Published:** 2022-01-20

**Authors:** Puhua Sun, Wenxiang Zhang, Xiao Yu, Jie Zhang, Ningkun Xu, Zhichao Zhang, Mengyuan Liu, Dongpei Zhang, Guangyu Zhang, Ziyuan Liu, Chaohe Yang, Wenjuan Yan, Xin Jin

**Affiliations:** ^1^ State Key Laboratory of Heavy Oil Processing, College of Chemical Engineering, China University of Petroleum, Qingdao, China; ^2^ Sinopec Research Institute of Safety Engineering, Qingdao, China

**Keywords:** hydrogenolysis, biomass, comparative study, assessment, glycerol, propylene glycol

## Abstract

Hydrogenolysis of glycerol to propylene glycol represents one of the most promising technologies for biomass conversion to chemicals. However, conventional hydrogenolysis processes are often carried out under harsh H_2_ pressures and temperatures, leading to intensive energy demands, fast catalyst deactivation, and potential safety risks during H_2_ handling. Catalytic transfer hydrogenolysis (CTH) displays high energy and atom efficiency. We have studied a series novel solid catalysts for CTH of glycerol. In this work, detailed studies have been conducted on energy optimization, tech-economic analysis, and environmental impact for both processes. The key finding is that relatively less energy demands and capital investment are required for CTH process. CO_2_ emission per production of propylene glycol is much lower in the case of transfer hydrogenolysis. The outcome of this study could provide useful information for process design and implementation of novel hydrogenolysis technologies for other energy and environmental applications.

**Graphical Abstract:**
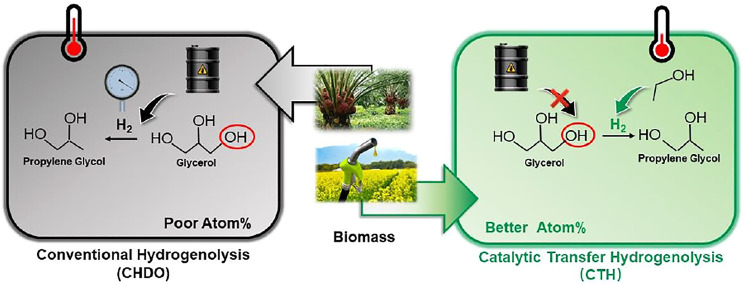
Table of Content Graph: Process analysis for conventional hydrogenolysis and transfer hydrogenolysis of glycerol reveals the energy, economic and environmental impact on biomass conversion.

## Introduction

Aqueous phase hydrogenolysis of bio-oxygenates provides a most promising technology for the production of various megaton chemicals from renewable feedstocks. (Wang et al., 2015a; Jin et al., 2019a; Park et al., 2021). In this context, hydrogenolysis of polyols to glycols and alcohols could offer alternative synthetic routes and alleviate the use of hydrocarbons for energy-intensive conversion processes. In particular, hydrogenolysis of glycerol, a bio-diesel by-product, can produce propylene glycol (PG), 1,3-propanediol, ethylene glycol (EG), as well as propanols for many downstream applications such as anti-freezes, polyesters, pharmaceuticals, and solvents. This is one of the most popular subjects which is under extensive studies in both academia and industry. (Haldar and Mahajani, 2004; Guo et al., 2009). However, hydrogenolysis of glycerol is often conducted under elevated temperatures and H_2_ pressures (>230°C, >4 MPa). Actually, this process is still heavily dependent on the use of fossil-derived H_2_, with cost-ineffective investment on H_2_ compression, recycling, and manufacture of process equipment. (Humbird et al., 2017; Freitas et al., 2018). It is true that renewable H_2_ (“green H_2_”) can now be produced from other feedstocks, e.g., electrolysis of water. But one should note that most hydrogenation plants have to be established adjacent to H_2_ source or pipelines, which could unfavorably increase operational and constructional cost for biorefineries. In addition, potential safety risk is another important factor hindering industrial implementation of this technology. Advanced aqueous phase hydrogenolysis technologies with low-carbon emission are highly demanded for future development of bio-refineries.

**FIGURE 1:**
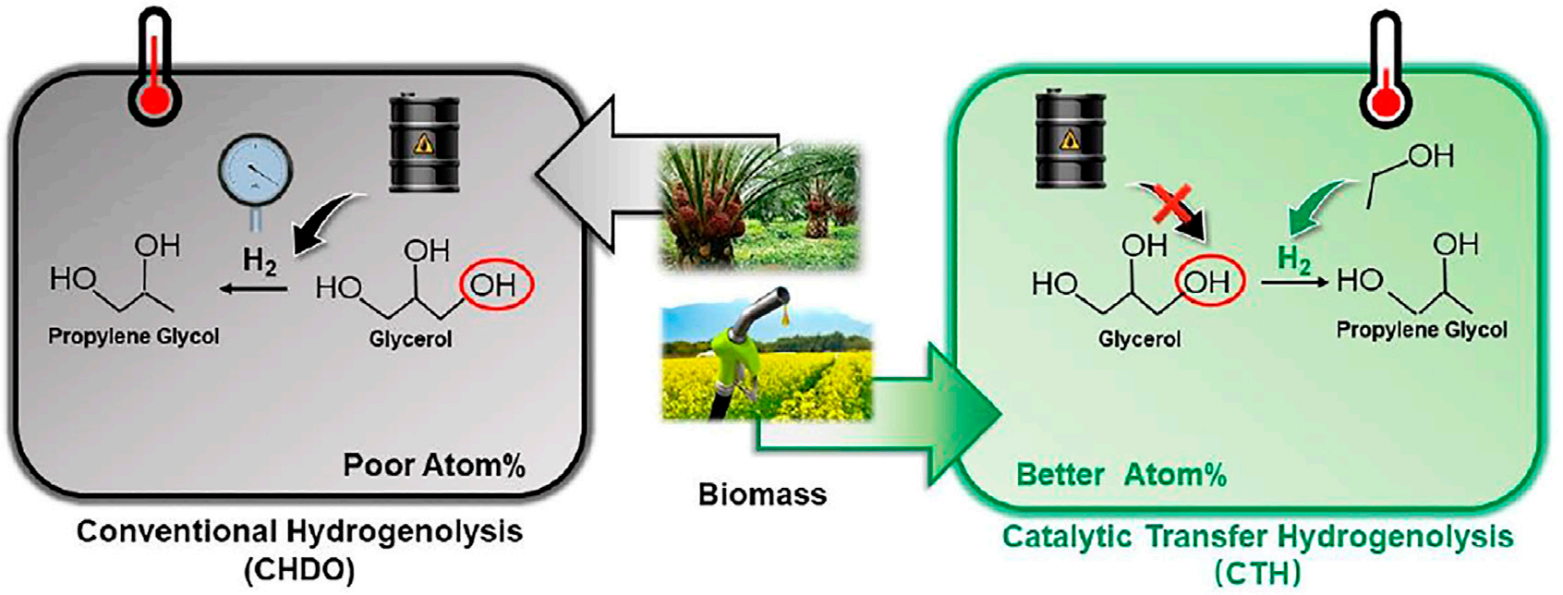
Conventional HDO (CHDO) and catalytic transfer hydrogenolysis (CTH).

Catalytic transfer hydrogenolysis (CTH), which takes use of renewable H-donors in liquid medium, is considered as one of the most economically and environmentally beneficial technique to substitute conventional hydrogenolysis processes (CHDO, Figure 1). (Jin et al., 2019b; Zhang et al., 2020a; Yin et al., 2020; Nie et al., 2021). Much milder reaction temperatures, higher intrinsic hydrogenolysis rates in aqueous medium, and lower operating pressures in the absence of externally added H_2_ make CTH processes more energy and atom efficient compared with CHDO processes. Extensive research efforts have been devoted to develop active and selective solid catalyst materials with bifunctional natures for both H_2_ generation and hydrogenolysis reactions. Pt-based (Tike and Mahajani, 2006; Falcone et al., 2015; Feng et al., 2015; Von Held Soares et al., 2017; Zhang et al., 2020b; Hu et al., 2020; Xia et al., 2020; Nie et al., 2021; Song et al., 2021), Cu-based (Nie et al., 2021; Feng et al., 2015; Wang et al., 2015b; Priya et al., 2016; Feng et al., 2011; Kant et al., 2017; Vasiliadou and Lemonidou, 2013; A et al., 2017), and Pd-based (Tike and Mahajani, 2006; Feng et al., 2015; Mauriello et al., 2015; Sun et al., 2017; Shafaghat et al., 2019; Xia et al., 2020; Song et al., 2021) catalysts have been proposed and investigated with respect to structure-performance relations. Those studies have confirmed that CTH of glycerol can be achieved under milder temperatures (<200^o^C) and pressures (inert <2 MPa), with remarkable atom efficiency for the synthesis of PG as the main product (Wang et al., 2013; Feng et al., 2015). Experimental studies have demonstrated the feasibility of CTH technique for catalytic upgrading of glycerol as well as other bio-oxygenates.

However, to our best knowledge, limited studies on energy, economic, and environmental assessment (E3A) have been carried out to evaluate the overall impact of CTH processes particularly for the production of PG from glycerol (D’Angelo et al., 2018; Gonzalez-Garay et al., 2017). This study is very important, as it will provide the overall energy efficiency, greenhouse gas emission, and economic advantages of CTH processes. More critically, the hot spot detected in E3A studies will also offer insights into process optimization in terms of energy and capital investment (D’Angelo et al., 2018; Arpia et al., 2021; Beneroso et al., 2017; Wicker et al., 2021; Xiang et al., 2014; Zaimes et al., 2015; Zhao et al., 2021) to further reduce the carbon-footprint and operating cost for facile production of PG.

However, to our best knowledge, no relevant reports have been published on comparative studies of energy, economy, and environmental assessment for both CHDO and CTH of glycerol. Therefore, in this work, we reported a detailed and comparative study on CHDO and CTH of glycerol to PG. Specifically, 1) energy consumptions for glycerol pretreatment, hydrogenolysis reactors, and separation/purification sections have been compared for both CHDO and CTH. Heat exchange network has also been optimized. 2) Economic analysis on how reduction of H_2_ compression contributes to overall economic improvement has been conducted. 3) Environmental analysis has also been carried out to estimate the CO_2_ emission and water discharge for both processes, respectively. Finally, both advantages and disadvantages for CTH of glycerol have been quantitatively discussed for future design of next-generation bio-refineries.

## Methodology

### Thermodynamic Model

In this work, the following chemicals have been incorporated for process simulation, water, glycerol, ethyl alcohol, propylene glycol (PG), propanol (PrOH), ethylene glycol (EG), acetol, cycloheptane, methanol (MeOH), ethanol (EtOH), acetic acid, CH_4_, H_2_, and CO_2_. Redlich-Kwong and NRTL models were adopted for simulation. Redlich-Kwong model was used to determine gas composition, while NRTL method was used to describe liquid phase (Rocha et al., 2014; Li et al., 2016; Liu et al., 2018). Since reaction and separation units involve gas-liquid and liquid-liquid multiphase system in this work, NRTL (non-random two liquid) model is supposed to be well predictive in determining the actual composition of various components (Poozesh et al., 2015; Li et al., 2016; Mirzaei et al., 2016; Xie et al., 2016; Hernández et al., 2018; Ma et al., 2018; Ma et al., 2019; Qin et al., 2019; Chen et al., 2020).

Redlich-Kwong and NRTL models are only suitable for polar and gas-liquid phase systems. Tech-Economic analysis has been focused on utilities and capital investment. There are many empirical parameters which may generate large errors. However, those errors are acceptable for engineering design.

### Technical Approach and Process Simulation

Both CHDO and CTH plant studied in this work consist of three main units: pretreatment unit, hydrogenolysis unit where glycerol is converted into PG and other co-products, and separation and purification unit. In this work, reaction results over Ni/Cu/TiO_2_ catalyst were used for simulation of CHDO process (Cai et al., 2018; Jin et al., 2019a; Jin et al., 2019b; Xia et al., 2020; Yin et al., 2020). Ni/Cu/TiO_2_ catalyst was selected for CHDO process because it displays leading performances in conversion and PG selectivity in literature. It has been a widely accepted catalyst material for numerous hydrogenation applications. Results obtained from PtFe-based catalyst, which were reported in recent work, were selected as the reaction model to simulate CTH process (Zhang et al., 2020a). Compared with other investigated catalysts for transfer hydrogenolysis reaction, PtFe-based catalyst displays much higher activity, although selectivity towards PG (58.7%) can be still improved in future studies.

**FIGURE 2:**
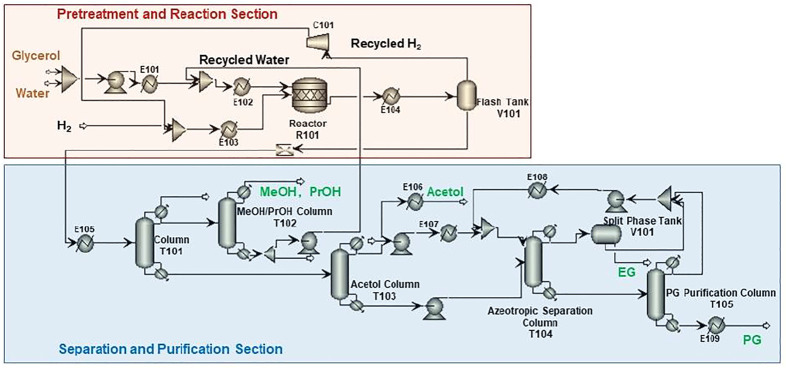
Process scheme for CHDO of glycerol.

Process scheme for CHDO of glycerol is presented in Figure 2. It is seen that this scheme consists of three major units, pretreatment unit and reaction unit, followed by separation and purification unit. Glycerol, water, and H_2_ are mixed with a ratio of 1:20:10 (molar) and fed to the reactor (R101 in Figure 2), where hydrogenolysis reaction is conducted under 230°C and 3.5 MPa H_2_ pressure. The main products include PG, PrOH, MeOH, and acetol, with chemoselectivity being 86.5, 1.5, 10.6, and 1.4%, respectively. The separation sequence for CHDO includes primary separation (T101), MeOH/PrOH separation (T102), acetol purification (T103), azerotropic separation (T104), and PG purification units (T105). Cycloheptane is used as azerotropic agent and recovered from T104 and T105 (Dohnal et al., 2014).

**FIGURE 3:**
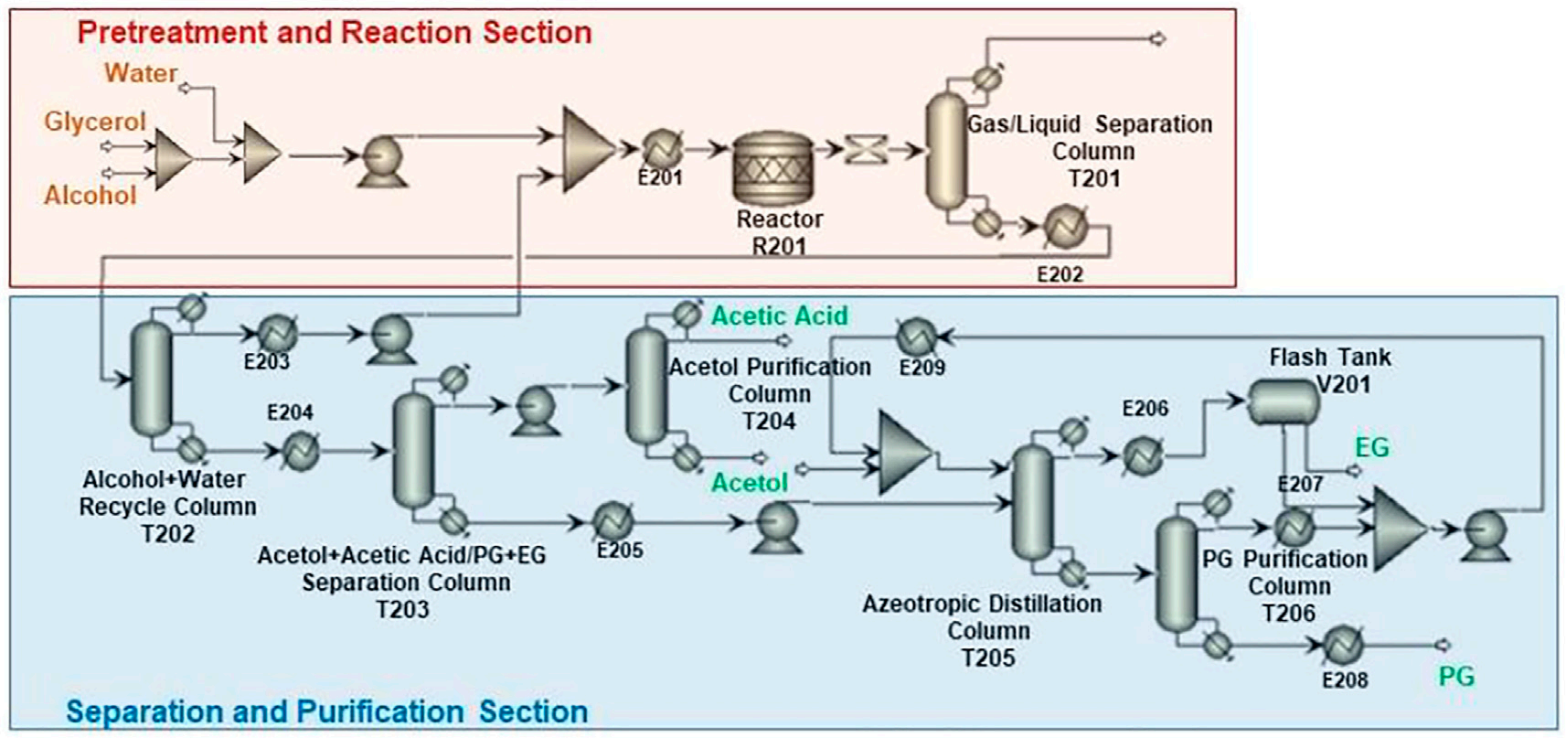
Process scheme for CTH of glycerol.

Figure 3 presents the process scheme for CTH of glycerol, which also consists of pretreatment unit, reaction unit, and purification unit. Glycerol is fed with EtOH (H-donor) as the feedstock into the reactor. Glycerol/EtOH/H_2_O (69.7/24.8/0.5 M ratio), together with recycling water, is pumped into reactor (R201 in Figure 3). The reaction effluent is first flashed in a Gas/Liquid Separation Column (T201) in this scheme to separate gas components such as trace amounts of H_2_ and CO_2_ from reaction mixture. EtOH and water are recycled into the reactor in Alcohol + Water Recycle Column (T202), in which the raw material could be saved. After being separated in Acetol + acetic acid/PG + EG Separation Column (T203), Acetol is purified in Acetol Purification Column (T204), while PG is purified in azeotropic distillation system including Azeotropic Distillation Column (T205) and PG Purification Column (T206).

### Reaction Scheme for CTH Process

Reaction parameters such as temperatures, pressures, and catalyst loading have been studied experimentally in our laboratory, as already reported previously (Jin et al., 2019a; Jin et al., 2019b; Zhang et al., 2020a; Zhang et al., 2020b; Yin et al., 2020). Conversion and selectivity data obtained from those reports were used as input for determination of configuration of reactor unit in simulation (Von Held Soares et al., 2017; Zhang et al., 2020a; Yin et al., 2020). As shown in Table 1, the reactions considered for this work include 1) dehydrogenation of EtOH, 2) dehydration of glycerol, and (3–4) hydrogenolysis of glycerol. More details of reactor design are presented in supporting information Reactor Design.

**TABLE 1:** Reactions of CTH scheme.

Main products	Selectivity (%)
PG	58.7
Acetic acid	13.9
Acetol	11.6
EG	5.1

### Models

Rstoic model was applied for the hydrogenolysis reactor. In separation and purification section, the main unit operations are fractionation and azeotropic distillation; thus, the RadFrac model was used. This model could be used for accurate calculation of each column (Humbird et al., 2017; Pla-Franco et al., 2019). Properties for major components were summarized in Supplementary Table S1, S2 for setting up separation sequences and further optimization. Aspen Energy Analyzer was used to carry out optimization of heat exchange network (refer to Supplementary Figure S5 for more details).

### Economic Analysis

In this part, calculation of investment, capital cost, and revenue was conducted for CHDO and CTH process. The capital cost for manufacturing and facilities is defined as the total direct capital cost (TDC) (Xiang et al., 2014; Zhou et al., 2019; Saavedra del Oso et al., 2020) which is estimated by the production capacity index approach, and it is defined as:

$$\text{TDC}=\sum_{i=1}^{m}C_{E,i}=\sum_{i=1}^{m}C_{basic}{\left(\frac{Q}{Q_{basic}}\right)}^{\alpha }f$$ (1)

where m is the total unit in process design, C_E,i_ is the capital cost of each process corresponding to actual capacity Q, C_basic_ indicates the capital cost with the base case capacity, α is the cost scale factor, and f is a comprehensive adjustment factor (Zhou et al., 2019; Cormos, 2021; Marchese et al., 2021). Q/Q_basic_ is 1/1 for both CHDO and CTH processes, the ratio of which was determined based on previous literature (Zhou et al., 2019; Cormos, 2021; Marchese et al., 2021). Details about equipment costs are presented in Supplementary Table S8, S9 of supporting information. Therefore, the indirect plant expense (IPE) is estimated as 30% of TDC, which consists of the capital costs for engineering and supervision, start-up capital, spares costs, construction expenses, contractor fees, and other contingency cost. TDC and IPE are combined to estimate the total plant capital cost (TPC) of CHDO and CTH scheme, which is defined as:

TPC=TDC+IPE (2)

The capital cost summary of CHDO and CTH scheme is shown in Table 5. More details are talked in results and discussion.

### Environmental Analysis

In this part, environment analysis is mainly focused on evaluating and comparing the environmental impacts of producing PG from CHDO and CTH processes. The environment analysis includes direct and indirect emission of CO_2_, the effluent discharge, the organic compounds, and equivalent greenhouse gas emissions in CHDO and CTH process. The quantitative analysis is reported according to million $ production (Saavedra del Oso et al., 2020).

As the main greenhouse gas (GHG), CO_2_ emissions can be calculated by the sum of direct emissions and indirect emissions. The direct emissions are mainly from reactions such as “C_3_H_8_O_3_+3H_2_O = 3CO_2_+7H_2_” and the process emissions, while the indirect emissions are caused by process energy production such as steam for heating and electricity for driving equipment.

## Results and Discussion

### General Information on CHDO Process

Prior to detailed comparative studies for both CHDO and CTH processes, it is important to set up the reference operating conditions for systematic investigation. Despite extensive studies on CHDO processes, there is lack of experimental kinetic data for the leading Ni/Cu/TiO_2_ catalyst in literature. As a result, the operating conditions for Ni/Cu/TiO_2_ catalyst in CHDO of glycerol cannot be optimized in this work. Such optimization would not be reliable without kinetic data under different temperatures and pressures from experimental studies. Therefore, the operating conditions Ni/Cu/TiO_2_ catalyst.

### Process Schemes

It is important to mention that H_2_ is recycled and compressed in this scheme (V101). To simplify the scheme, we have reduced the purification part for H_2_ recycling for the convenience of further cost estimation. It is also critical that, for CHDO process, we already found that recycling water is not economically viable as the cost for H_2_ purification. Those preliminary calculations showed that H_2_O recirculation and H_2_ purification are not expedient from the technological point of view, and therefore, they are excluded from the technological schemes.

Different from CHDO process, CTH of glycerol is composed of relatively simpler scheme. H_2_ compression unit is not required, as there is no external H_2_ added in CTH of glycerol. However, glycerol should be fed with EtOH (H-donor) as feedstock into the reactor. Glycerol/EtOH/H_2_O (69.7/24.8/0.5 M ratio) is pumped into reactor (R201 in Figure 3) for CTH reactions. This ratio is pre-determined based on our previous report for experimental studies. The reaction effluent is first flashed in a gas/liquid separation column (T201), then gas components such as trace amounts of H_2_ and CO_2_ from reaction mixture are separated. Light components including CH_4_, H_2_, CO_2_, and trace MeOH were sent to furnace for combustion. Another difference for CTH of glycerol, compared with CHDO process, is the separation unit. In CTH of glycerol, the main products, as described in previous sections, include PG, EG, acetol, acetic acid, and MeOH, leading to distinct separation scheme for the proposed process. In particular, alcohol + water mixture (EtOH/MeOH/H_2_O: 29/4.6/66.4 M ratio) is recycled back with fresh feedstock with Glycerol/EtOH/H_2_O stream. The remaining unreacted alcohols can be used as H-donor for fresh feedstocks. For CTH process, dehydration of glycerol leads to the formation of acetol, while dehydrogenation of EtOH eventually generates acetic acid as the final product. Acetol and acetic acid are separated from PG and EG in T203, while acetol could be further obtained in T204 with 99.95% purity. Similar to CHDO scheme, PG and EG are separated using azeotropic distillation sequence (T205-T206, refer to supporting information for more details).

Based on description presented above, it is clear that, for CTH of glycerol, H_2_ cycling and product separation units are completely different from the conventional one. Therefore, we were motivated to further study the advantages and possible disadvantages of CTH technology for glycerol conversion in the following sections.

### Detailed Studies on Process Parameters for Both Processes

**FIGURE 4:**
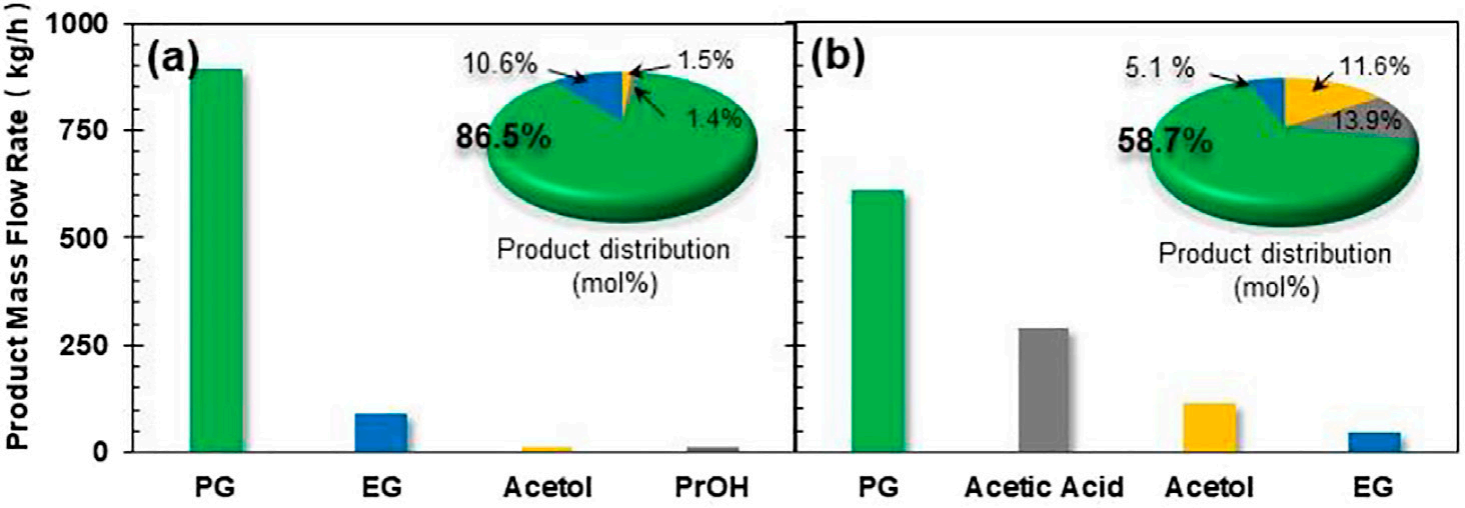
Product distribution and mass flow rate for **(A)** CHDO and **(B)** CTH of glycerol.

Prior to detailed investigation on the influence of process parameters on productivity and energy/capital costs, we first compared the product flow rate and composition for CHDO and CTH processes. The mass flow rates for PG, EG, acetol, and PrOH are 893.4 kg/h, 89.3 kg/h, 14.1 kg/h, and 12.3 kg/h, respectively, while CTH of glycerol leads to 606.3 kg/h, 42. kg/h, and 116.6 kg/h for those products. The product distribution (carbon-based selectivity, 58.7%) (Zhang et al., 2020a; Yin et al., 2020) for the two processes has also been presented in inset of Figure 4. It is found that, for CHDO process, the main products include PG (86.5%) and EG (10.6%), with trace amounts of acetol and PrOH as co-products. Interestingly, for CTH of glycerol using EtOH as H-donor, the main products are PG (58.7%), acetic acid (13.9%), and acetol (11.6%). Clearly, the product distribution from the two processes is distinct from each other. This is because that for CTH process, H_2_ generation from EtOH as H-donor leads to the formation of acetic acid in the reaction medium, while conversion of glycerol undergoes dehydration and hydrogenolysis steps, forming acetol and PG as intermediate and final products, respectively.

**FIGURE 5:**
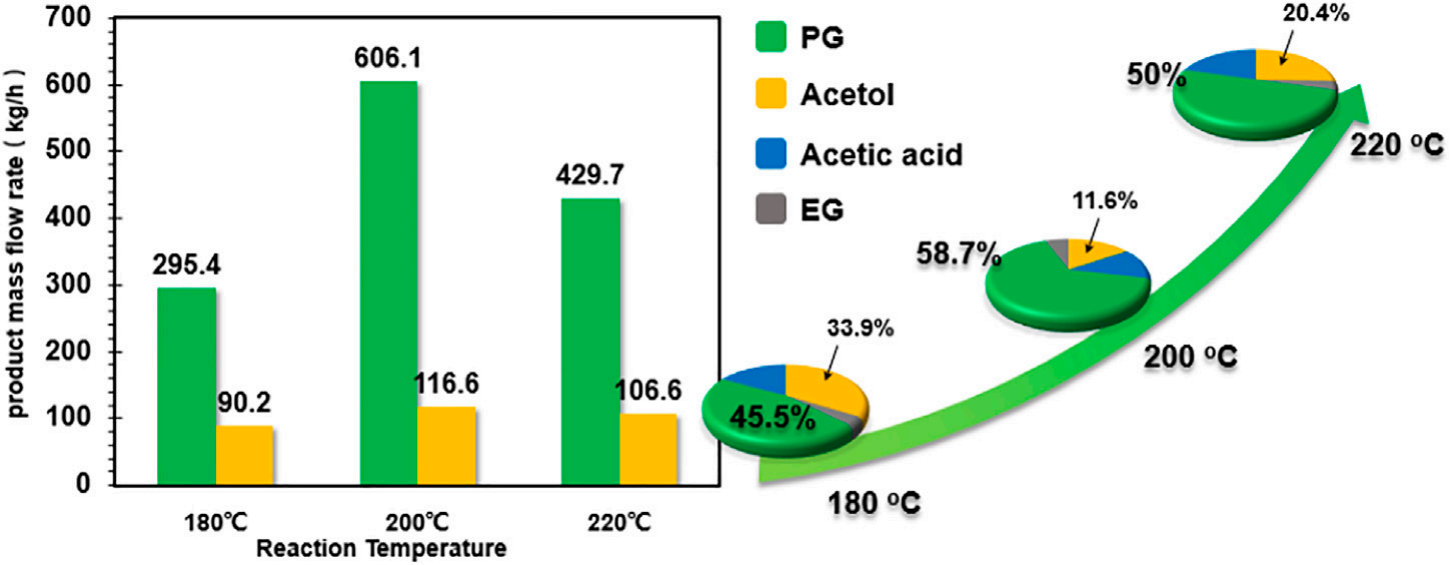
Influence of reaction temperature on product flow rate and distribution.

The influence of temperature on product distribution and product mass flow rate is presented in Figure 5. It is observed that increasing reaction temperature from 180°C to 200°C leads to improved selectivity to PG from 45.5 to 58.7%, with decreasing selectivity for acetol from 33.9 to 11.6%. However, further increasing temperature to 220°C causes significant degradation reactions with CH_4_ and CO_2_ as by-products. As a result, the overall product mass flow rates for PG and acetol are much lower than that at 200°C.

**FIGURE 6:**
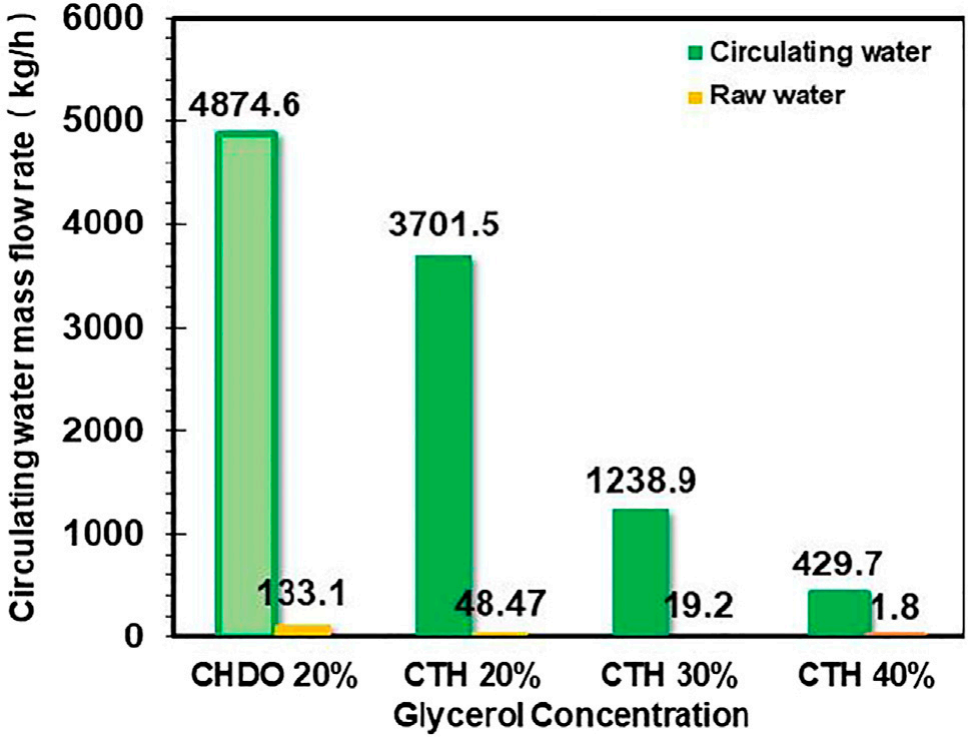
Influence of glycerol concentration in aqueous feed on product flow and distribution.

We also studied the influence of glycerol concentration on the amount of total recycled water for CTH scheme. It is found from Figure 6 that increasing glycerol concentration from 20wt% to 40wt% in the feedstock leads to significant changes of water consumption. In particular, the CTH process with 20wt% glycerol concentration in the feedstock demands 2014.2 kg/h and 1735.8 kg/h of recycling water and additional water input, respectively. As glycerol concentration enhanced to 30wt%, we confirmed that the water demands for CTH process have been decreased dramatically, down to 1,238.9 kg/h and 19.2 kg/h, respectively, for recycling water and additional water input.

In this part of work, simulation of glycerol concentration effect on CHDO process could not be conducted due to lack of kinetic data in literature. It would be very useful to compare the concentration effect for both schemes, providing more critical insights into the sustainability of CHDO and CTH processes. Therefore, 20wt% glycerol concentration was still used for further comparison of two schemes to ensure consistency in processing capability of the two technologies.

It is also important to mention that the water generated in both CHDO and CTH meets the environment regulation for discharge. Therefore, CTH processes with much lower water demand is more favorable for lowering the overall cost of water treatment.

Based on the comparison shown above, it is clear that CTH process displays the following advantages:

1) Higher atom efficiency for value-added PG and acetol products. Considering the use of external pressurized H_2_ with high H_2_/glycerol ratio (10), for CHDO processes, utilization of stoichiometric liquid H-donors exhibits much better overall atom economics.
2) Lower water input. Based on Figure 6, the water demands for recycling and additional input are approximately 74.8% compared with CHDO process with 20wt% glycerol concentration in the feedstock.

The interesting results in Figure 6 provide further insights into nature of reaction network for both processes. For CHDO process, water is solvent and a by-product, while water is both reactant and product for CTH scheme. This is the reason that CHDO process needs additional water to provide reaction medium.

In the following sections, we will further discuss the energy demands for CHDO and CTH processes, which provide important evaluation for the economics and profit analysis.

### Influence of Glycerol Concentration and Temperature for CTH Process

Glycerol concentration will influence the reaction kinetics as well as separation cost. This is because glycerol concentration will influence the amount of recirculating water as well as the heat duty for each of the downstream separation column. In this section, energy requirement for CHDO and CTH under identical glycerol concentration will be compared prior to detailed investigation on how glycerol concentration in the feed affect energy cost for CTH scheme. The amount of water (solvent) used in this system under different glycerol concentration varies significantly from 20wt% to 40wt%, as already seen in Figure 6. In particular, altering glycerol concentration from 20wt% to 40wt% not only modifies the intrinsic conversion rate of glycerol in the presence of PtFe/Y catalysts but also changes the conversion rate of H-donor (EtOH) in aqueous medium. Increasing glycerol concentration undoubtedly decreases the overall water consumption in CTH process, thus further leading to lowered energy input. As found in Table 2, the energy requirement for R201, T201-206 is 430.4 kW, 14,631.3, 21308.9, 614.7, 261.2 kW, 5,501.5 kW, and 173.9 kW, respectively, contributing to a total of 42,822 kW under this scenario. Significantly, when glycerol concentration enhanced up to 40wt%, the energy duty for R201, T201, and T202 has been lowered by almost 30, 23, and 54%. This is because higher glycerol concentration actually lowers the energy input for heating up the feedstock, as well as the separating cost of water from reaction mixture. This is one of the key findings in the work.

**TABLE 2:** Energy consumption of main equipment in CTH process at different concentration of glycerol.

Three different concentration of glycerol	20wt%	30wt%	40wt%
Equipment			
R201 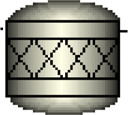	430.4	352.3	300.8
Catalytic transfer			
Hydrogenolysis Reactor			
T201 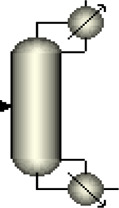	14,631.3	12,310.3	11,241.5
Gas/Liquid Separation			
Column			
T202 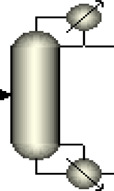	21,308.9	10,510.7	9,718.4
Alcohol + Water			
Recycle Column			
T203 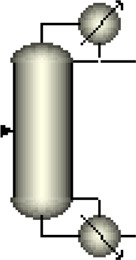	614.7	609.4	605.4
Acetol + acetic acid/PG + EG			
Separation Column			
T204 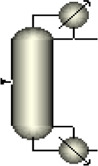	261.2	255.4	252.5
Acetol Purification			
Column			
T205 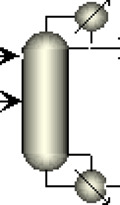	5,501.5	5,560.5	5,669.5
Azeotropic Distillation			
Column			
T206 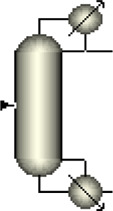	173.9	168.3	175.9
PG Purification			
Column			
Total Heat Demand (kW)	42,821.9	29,766.9	27,964.1

**FIGURE 7:**
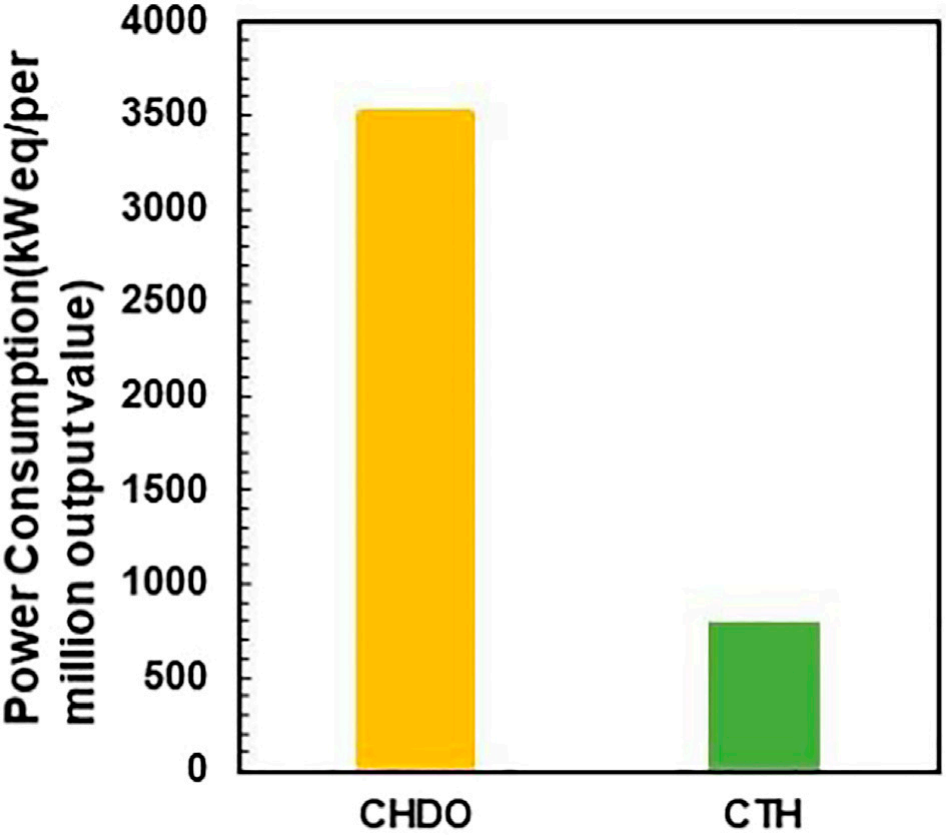
Power consumption of CHDO and CTH scheme.

The effect of reaction temperature on the energy input for pretreatment and reaction sections, as well as separating and purification sections was also studied in the following sections. It is found in Table 3 that, increasing reaction temperature from 180 to 220°C, energy input for R201 has been enhanced by almost 9-fold. Interestingly, the energy cost for T201-T206 does not vary significantly as expected. However, it is important to mention that changing temperature obviously modifies the reaction rate for both glycerol and EtOH, as well as product distribution. Therefore, the product composition in R201 is significantly different in all three cases in Table 3 (180°C, 200°C, and 220^o^C). Detailed information on product composition has already been shown in Figure 5. Overall, increasing reaction temperature results in a slight enhancement in energy consumption from 28,116 kW to 31,259 kW. The power consumption for both CTH and CHDO has been shown in Figure 7.

**TABLE 3:** Energy consumption of main equipment in CTH process at different concentration of glycerol.

Three different reaction of temperature	180°C	200°C	220°C
Equipment			
R201 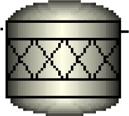	95.9	352.3	870.5
Catalytic transfer			
Hydrogenolysis Reactor			
T201 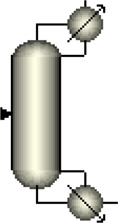	10.511.4	12,310.3	11,794.3
Gas/Liquid Separation			
Column			
T202 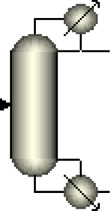	11,010.5	10,510.7	12.028.2
Alcohol + Water			
Recycle Column			
T203 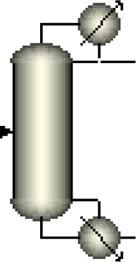	756.1	609.4	707.4
Acetol + acetic acid/PG + EG			
Separation Column			
T204 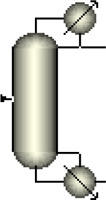	364.3	255.4	359.9
Acetol Purification			
Column			
T205 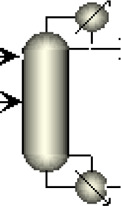	5,214.2	5,560.5	5,331.9
Azeotropic Distillation			
Column			
T206 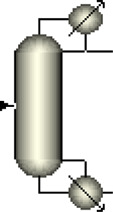	163.5	168.3	166.4
PG Purification			
Column			
Total Heat Demand (kW)	28,115.8	29,766.9	31,258.5

### Energy Analysis for Both Schemes

We have selected 20wt% glycerol concentration and 200°C as the reference condition for further process analysis. Energy analysis was conducted to reveal the energy consumption for each unit operation in both CHDO and CTH processes. Specifically, Table 4 presents the energy summary for material pretreatment, hydrogenolysis, and separation sections in both schemes. It is clear that material pretreatment for CTH process displays slightly lower energy consumption compared with conventional unit (entry#1). Most importantly, reaction unit for CTH shows significantly lower energy requirement (430.4 kW), compared with CHDO (2,247.4 kW). This is because operation temperatures and pressures for CTH process are 200°C and 3 MPa, respectively, which are milder than CHDO scheme (230°C, 3.5 MPa).

**TABLE 4:** Energy summary of CHDO and CTH scheme.

Process		CHDO (kW)		CTH (kW)	
Energy consumption					
1	Pretreatment Section	175.1 kW		149.5 kW	
2	Reaction Section	2,247.4 kW		430.4 kW	
**3**	Separating and Purification Section	T101^a^	39,398.0 kW	T201^#^	14,631.3 kW
		T102^a^	1,742.6 kW	T202^#^	21,308.9 kW
		T103^a^	332.5 kW	T203^#^	614.7 kW
		T104^a^	7,019.5 kW	T204^#^	261.2 kW
		T105^a^	1,024.5 kW	T205^#^	5,501.5 kW
		V101^a^	80.3 kW	T206^#^	173.9 kW
		Sum	49,597.4 kW	Sum	42,491.5 kW
4	Total Consumption	52,019.9 kW		43,071.4 kW	

a T101, Column *T102 MeOH/PrOH column; *T103, Acetol Column *T104 Azeotropic Separation Column; *T105, PG purification column; *T201, Gas/Liquid Separation Column; *T202, Alcohol + Water Recycle Column; *T203, Acetol + acetic acid/PG + EG separation column; *T204, Acetol Purification Column; *T205, Azeotropic Distillation Column; *T206, PG purification column.

For separating and purification sections, we have found that energy requirements for CHDO and CTH schemes are again different from each other. In CHDO process, water, MeOH, and PrOH were obtained from the overhead of T101 column, while the mixture from the bottom of this column contained PG, EG, acetol, and small amounts of water (4wt%). Water has been obtained from the bottom of T102 and recycled back to the mixer in the pretreatment and reaction section, with MeOH/PrOH obtained from overhead of T102. Acetol of 92wt% purity can be produced from T103 as one of the co-products from CHDO process. PG and EG are separated in T104 with cycloheptane as azeotropic agent. EG of 99.97wt% purity can be obtained from T104, while PG of 99.92wt% can be produced from T105. Clearly, the heat duty for T101 is the highest compared with other columns in CHDO scheme. The azeotropic unit consisting of T104 and T105 demands a total energy input of 8,044 kW.

Since the product distribution for CTH is different from that of CHDO, the separating and purification scheme for CTH varies significantly. In particular, T201 separates light gases such as H_2_ (7wt%), CH_4_ (9wt%), CO_2_ (79.8wt%), and MeOH (3wt%) from the product mixture. Alcohol and water mixture was produced from the top of T202 and recycled back to the mixer in hydrogenolysis unit.

Acetol and acetic acid were separated from PG and EG in T203, while purified acetol of 99.9wt% as the highly valuable co-product can be obtained from bottom of T204. Acetic acid of 97wt% purity (containing 0.2wt% acetol and ∼2.8wt% water as impurities) was produced from the top of T204. Similar to CHDO process, PG and EG were also separated *via* azeotorpic distillation, in T205 and T206 columns. It can be seen that alcohol and water recycle unit (T202) demands significantly high energy input in CTH scheme, while for CHDO process, separating water/MeOH/PrOH from product mixture needs remarkably high energy input (T201). The overall energy requirement for purification of acetol, PG, and EG is relatively lower in CTH process compared with CHDO scheme. As a result, the energy requirement for purification unit is 49,597 kW and 42,491 kW for CHDO and CTH, respectively. Considering the advantages of milder operation temperatures and pressures in CTH process, the total energy for the proposed process is 17% lower than CHDO scheme.

### Capital Cost and Revenue

Based on the detailed analysis shown above, we further conducted techno-economic analysis for CHDO and the proposed CTH process, with regard to energy consumption and product distribution. The total number of equipment is summarized in Supplementary Table S4. It is found that the overall number of equipment is similar in both schemes, except for flash tank and compressor. This is because CTH process does not require H_2_ compressor at all, while in CHDO scheme, H_2_ compressor is a critical unit to recycle excess H_2_ back to the reaction unit.

**FIGURE 8:**
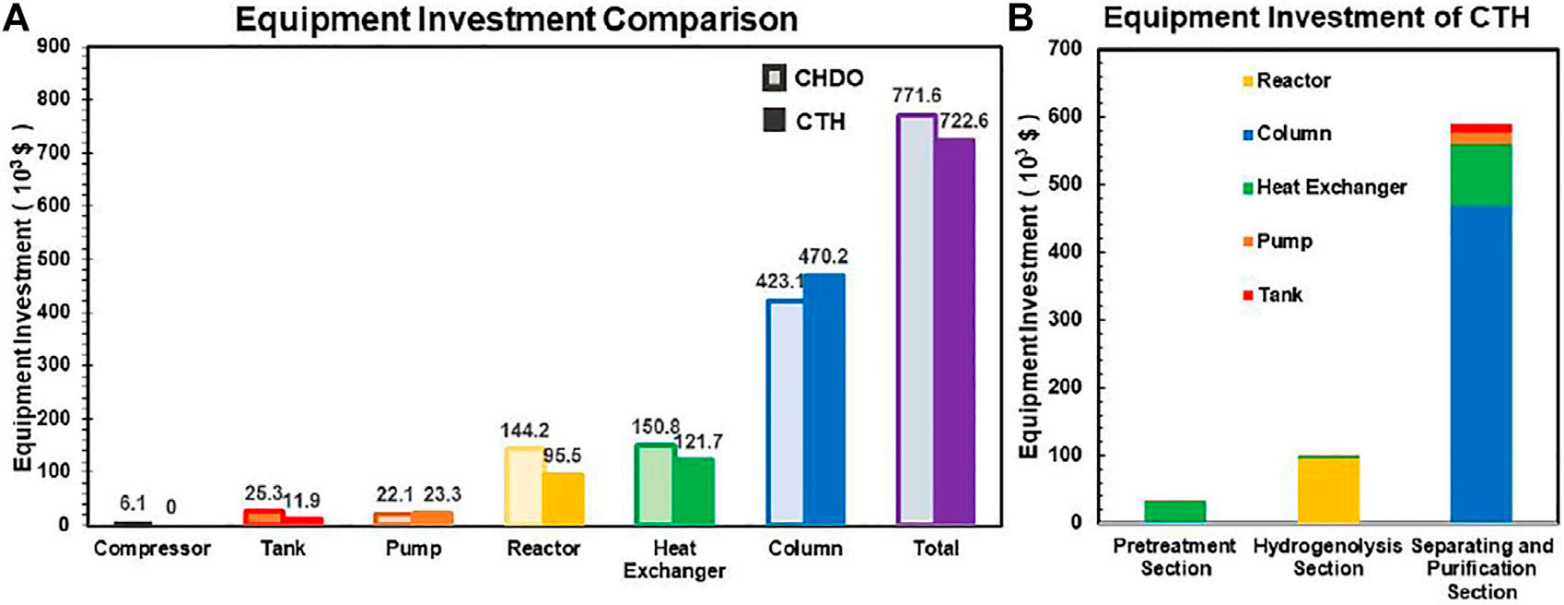
Equipment investment for CHDO and CTH processes **(A)**. Detailed capital cost for different unit operations in CTH process **(B)**.

Detailed capital investment for CHDO and CTH processes is compared in Figure 8A. It is found that the investment for tanks and pumps is similar for both processes. However, due to relatively lower operating pressure in CTH process, the cost for reactor and heat exchangers is lower than that of CHDO. Another important point is that CTH process requires relatively higher investment for separation scheme. This is because several reactions are involved in CTH scheme; thus, additional co-products such as acetic acid and acetol are produced. As a result, the cost for column is slightly more expensive. The overall equipment investment for CTH is more cost-effective than CHDO process. The capital cost of CHDO and CTH schemes is summarized in Table 5.

**TABLE 5:** Capital cost of CHDO and CTH scheme.

Items [10 (Jin et al., 2019a) $]	CHDO	CTH
Total Direct Cost (TDC)	1,234.6	1,155.4
Indirect Plant Expenses (IPE)	370.4	346.6
Total Plant Capital Cost (TPC)	1,605	1,502

It is important to point out that, since CTH process does not involve use of pressurized H_2_, the maintenance and safety cost will also be much lower compared with CHDO process. This is because that, in CTH scheme, reactor is conducted under much milder temperature and pressure. As a result, H_2_ compressor is not needed, with reduced operation cost.

In addition, CTH process produces other co-products including acetol, acetic acid, and EG; therefore, three additional storage tanks are needed. However, the overall cost for the three tanks is obviously lower than H_2_ tank and compressor.

Detailed cost assignment for different unit operations in CTH process is presented in Figure 8B. The key finding in this part is that separation is the main cost contributor in this scheme, where investment for both heat exchangers and columns accounts for as high as 81.6% in CTH process.

According to our detailed simulation on CHDO and CTH processes, the sale revenue for both processes is listed in Table 6. We further compared the revenue for various different products including EG, acetic acid, PG, and acetol. Due to the relatively lower product flow rates in CTH scheme, the sales revenue is slightly lower than that of CHDO. However, the lost revenue is trade off by the remarkable price for acetol product. Therefore, the overall revenue is approximately 3-fold higher in CTH scheme.

**TABLE 6:** Sale revenue for CHDO and CTH process.

Scheme	CHDO	CTH			
Product	PG	PG	Acetol	EG	Acetic acid
Annual Output (ton/a)	7,141.2	4,850.3	927.2	343.7	2,316.6
Purity (wt%)	99.9	99.9	99.1	99.9	99.5
Sales Revenue (10 Jin et al. (2019a) $)	14,830	10,080	43,400	186	1,070
Sales Total (10 Jin et al. (2019a) $)	14,830	54,736			

### Environmental Assessment

The novelty of transfer technologies has been demonstrated in numerous previous work (Sawadjoon et al., 2013). However, quantitative assessment for environmental impact of conventional and the proposed new process has not been investigated comprehensively in current literature. Therefore, in this section, detailed analysis for CHDO and CTH will be conducted and the results will be discussed systematically.

#### Boundaries

**FIGURE 9:**
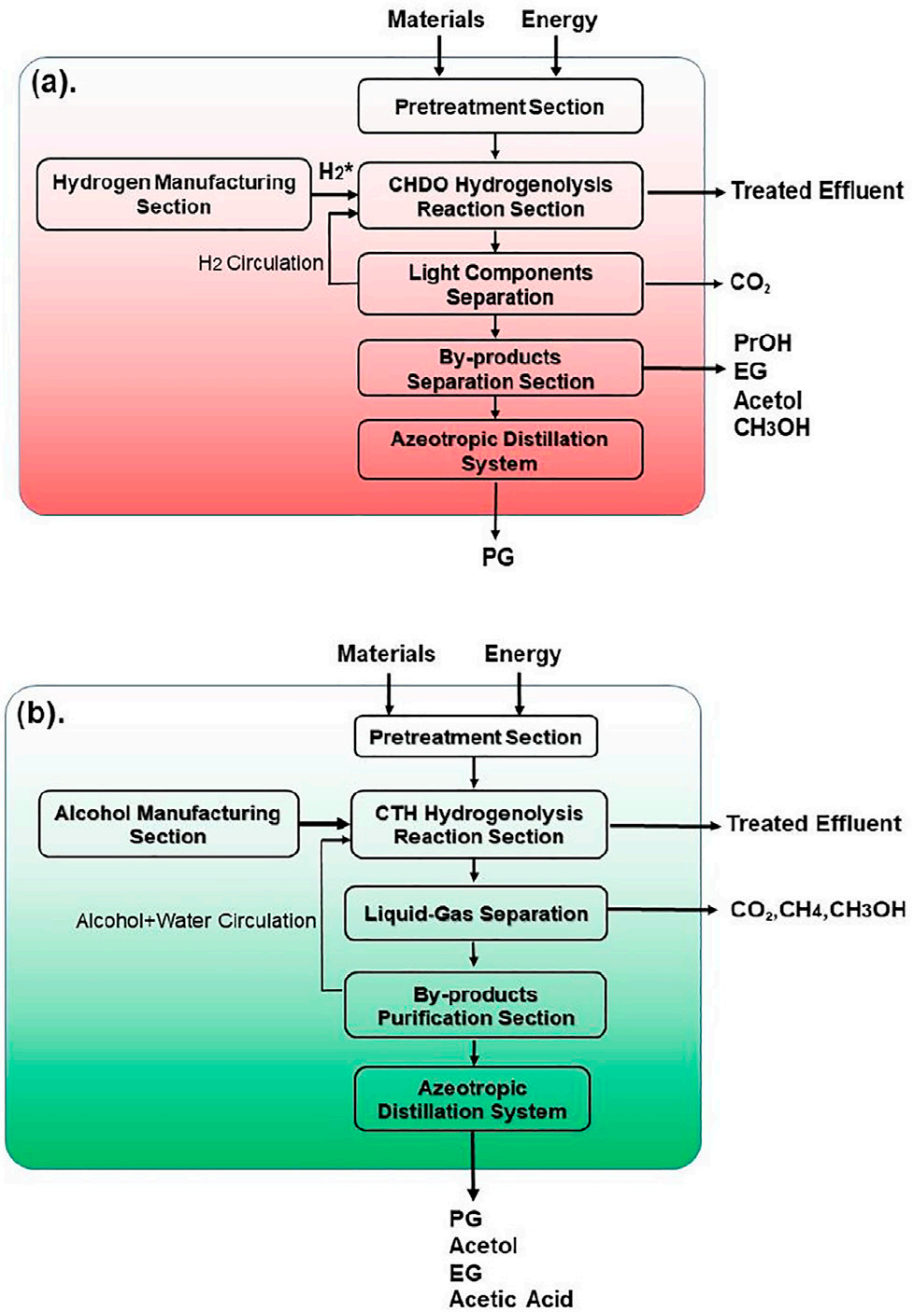
The system boundaries for **(A)** CHDO and **(B)** CTH processes.

The boundaries for environmental analysis for both processes are presented in Figure 9. For CHDO process (Figure 9A), the inlet materials include glycerol and H_2_. Actually, H_2_ is industrially derived from fossil fuels using coal or natural gas as the starting material. Therefore, the environmental impact from fossil-H_2_ generation is also included in this assessment. The leaving materials are mainly consisting of the oxygenate products and waste gases, such as PG, PrOH, EG, acetol, MeOH, and CO_2_. Differently, the entering materials for CTH process (Figure 9B) are mainly glycerol and EtOH, the latter of which can be also derived from bio-substrates. It is important to mention that CTH scheme does not require input of fossil-H_2_. Due to the complexity of reaction network in CTH process, the by-products are yet different from CHDO. For example, CH_4_ and acetic acid are main by-products (co-products). The leaving materials in CTH scheme also include PG, acetol, and EG, similar to CHDO process (Garcia-Herrero et al., 2016; Sternberg et al., 2017).

#### CO_2_ Emission per PG Production

Based on process simulation, it is clear that productivity of PG in CTH process is relatively lower compared with CHDO process, due to the co-production of highly valuable acetol. To quantify the CO_2_ emission, it is important to normalize based on PG production for comparative studies of CHDO and CTH processes.

**FIGURE 10:**
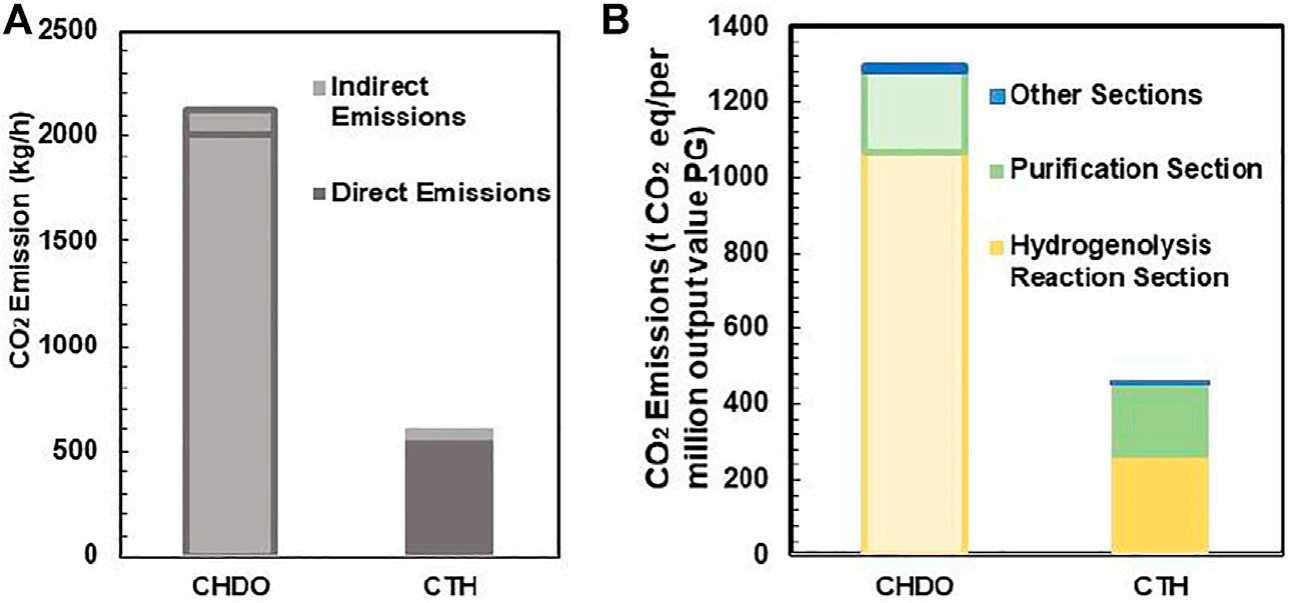
The direct and indirect emission of CO_2_ in CTH and CHDO **(A)**. Equivalent greenhouse gas emissions of CHDO and CTH **(B)**.

Emission of CO_2_ and effluent discharge will be assessed in this section for both processes (Sternberg et al., 2017). The results are presented in Figure 10. It can be seen from Figure 10A that the sum of indirect and direct CO_2_ emission for CHDO is almost 5-fold higher than CTH process. Direct CO_2_ emission is defined as formation of this product from hydrogenation unit and post-treatment of CO as by-product. Therefore, it is not surprising that the direct CO_2_ emission from CHDO is much higher than that of CTH. Indirect CO_2_ emission include the CO_2_ output from process utilities. It is clear that both CHDO and CTH only discharge small amounts of indirect CO_2_.

Effluent discharge is also compared in this work. Again, the discharge is approximately 73 kg/h for CHDO, which is almost 5-fold higher than CTH scheme. This is because that, in CHDO process, water is the product rather than reactants. Therefore, significant amounts of wastewater are generated under this condition. In CTH process, water is one of the important reactants for H_2_ generation; as a result, the utilization efficiency is very high. Thus, wastewater discharge is minimized under this circumstance.

**FIGURE 11:**
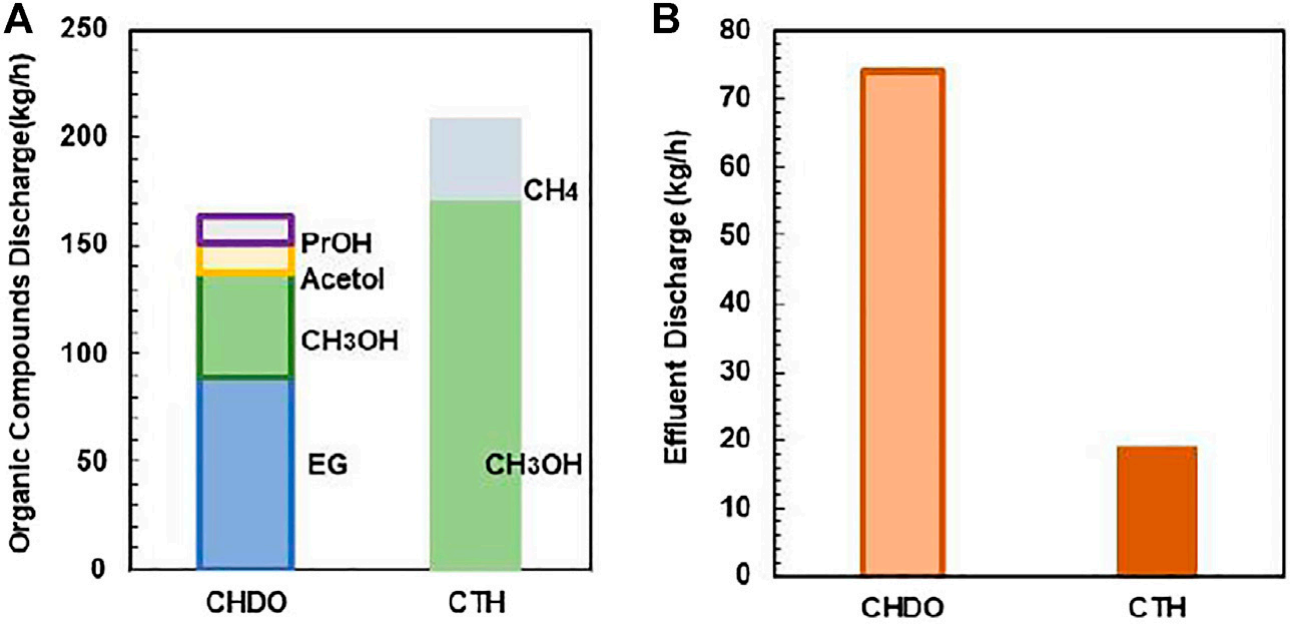
The composition of the organic compounds of CHDO and CTH **(A)**. The effluent discharge of CHDO and CTH **(B)**.

The discharge for other organic compounds has also been compared (Figure 11). Due to the formation of various other co-products such as CH_4_, acetic acid, and MeOH, the discharge of organic products is higher in CTH process. This is because we have not considered the purification part for the two chemicals, which can be further improved in future work.

The CO_2_ emission per million value was eventually compared for CHDO and CTH. It is very critical to mention that acetol is not the hydrogenation product, although the value of which contributes significantly to the final sales revenue. CO_2_ emission per million value should be evaluated only for hydrogenation product (Xiang et al., 2014; Zhou et al., 2019). In this work, PG is actually the target product for both CHDO and CTH processes. The data presented in Figure 10 only reflect the CO_2_ footprint for PG formation. According to the analysis, CO_2_ output is dramatically higher in CHDO scheme, while that in CTH scheme has been reduced by 71%. Further detailed analysis revealed that, actually contribution of CO_2_ output from purification and pretreatment section is similar for CHDO and CTH. However, CHDO will release significant amounts of CO_2_ owing to the harsh operating temperature and H_2_ pressure. According to the environmental impact analysis, it is clear that our proposed transfer hydrogenolysis technology imposes less carbon footprints in comparison with CHDO process, thus displaying great potentials for large scale implementation in bio-refineries.

## Conclusion

In this work, we have conducted detailed analysis for both conventional hydrogenolysis (CHDO) and catalytic transfer hydrogenolysis (CTH) of glycerol processes. Process scheme for both processes has been discussed with respect to energy consumption, productivity, and product distribution. The key finding in this part is that total energy input for CTH of glycerol is approximately 83% of CHDO process. Detailed economic analysis has demonstrated a total reduction of 7% for total investment in CTH scheme, owing to simplification of H_2_ handling equipment. Furthermore, influence of two critical parameters, glycerol concentration and reactor temperature on energy consumption, recycling water, and productivity for each key product, has been further discussed in detail. As one of the key findings, catalytic transfer hydrogenolysis process demand less energy input compared with conventional processes. More importantly, economic analysis confirmed the advantage of transfer hydrogenolysis due to the reduction of H_2_ recycling and compression units. Owing to the elimination of natural gas-derived H_2_ source, the overall CO_2_ emission in transfer hydrogenolysis has been reduced by more than 71%. Further analysis confirms that a remarkable improvement for CTH over CHDO with a total of 64% reduction in CO_2_ emission (per $ per production of propylene glycol) has been achieved.

**FIGURE 12:**
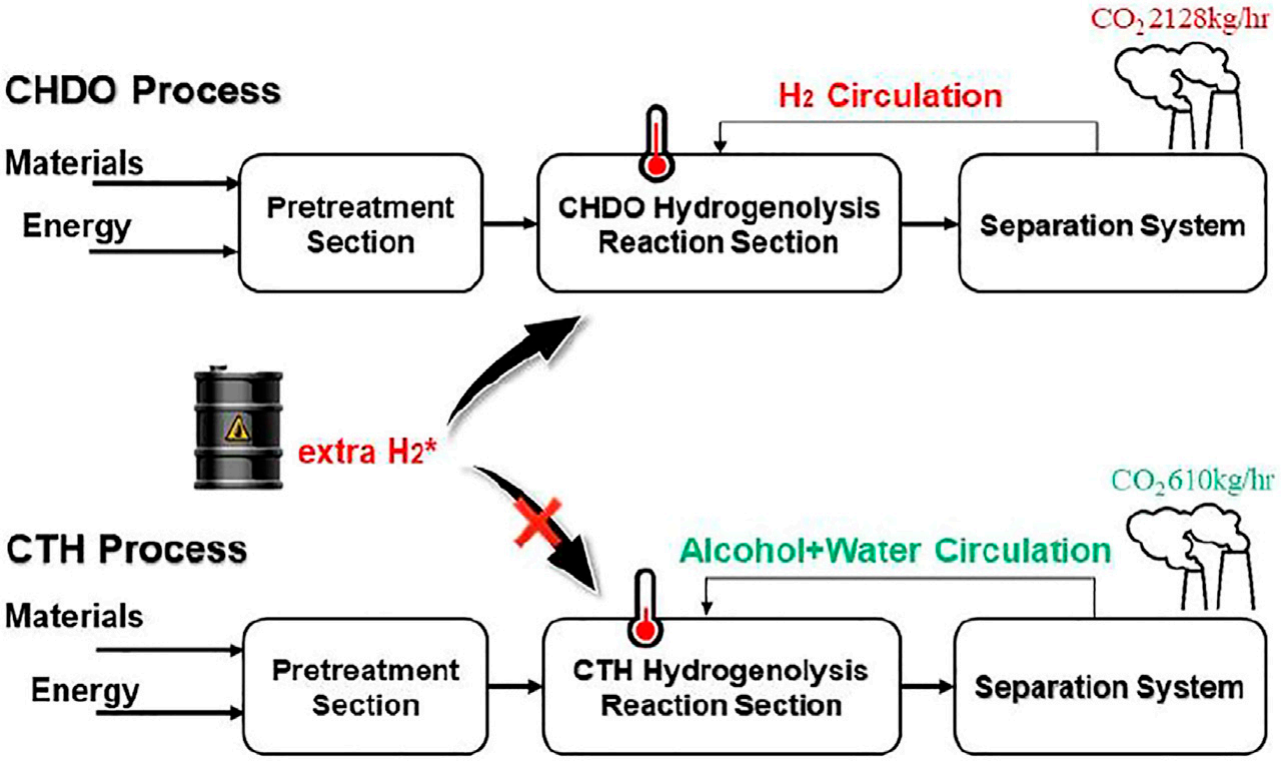
The advantages of CTH compared with CHDO.

This work has clearly demonstrated the advantages of transfer hydrogenolysis of glycerol to propylene glycol over PtFe-based catalysts. This work will be useful for future process development and implementation of transfer hydrogenolysis technologies in next-generation bio-refineries. The advantages of CTH compared with CHDO are shown in Figure 12.

## Supporting Information

Additional information on process schemes, equipment design, economic analysis, and heat transfer optimization are available in supporting information.

## Data Availability

The original contributions presented in the study are included in the article/Supplementary Material. Further inquiries can be directed to the corresponding authors.
