# Supplementary material for: Hydrogenolysis of Glycerol to Propylene Glycol: Energy, Tech-Economic, and Environmental Studies
Source: Front Chem. 2022 Jan 20;9:778579. doi: 10.3389/fchem.2021.778579 (PMC8811453; doi:10.3389/fchem.2021.778579)
Supplement: Supplementary file 1 [file Table2.DOCX]

**Supporting Information**

**Hydrogenolysis of Glycerol to Propylene Glycol: Energy, Tech-Economic and Environmental Studies**

Puhua Sun^1^, Wenxiang Zhang^1^, Xiao Yu^1^, Jie Zhang^1^, Ningkun Xu^1^, Zhichao Zhang^1^, Mengyuan Liu^1^, Dongpei Zhang^1^, Guangyu Zhang^2^, Ziyuan Liu^1^, Chaohe Yang^1^, Wenjuan Yan^1*^, Xin Jin^1*^

^1^State Key Laboratory of Heavy Oil Processing, College of Chemical Engineering, China University of Petroleum, No. 66 Changjiang West Road, Qingdao, Shandong Province 266580, China

^2^Sinopec Research Institute of Safety Engineering, No. 218 Yanan San Rd, Qingdao, Shandong Province 266580, China

Corresponding authors:

Xin Jin (jamesjinxin@upc.edu.cn), Wenjuan Yan (wenjuanyan@upc.edu.cn)

#### Streams Information


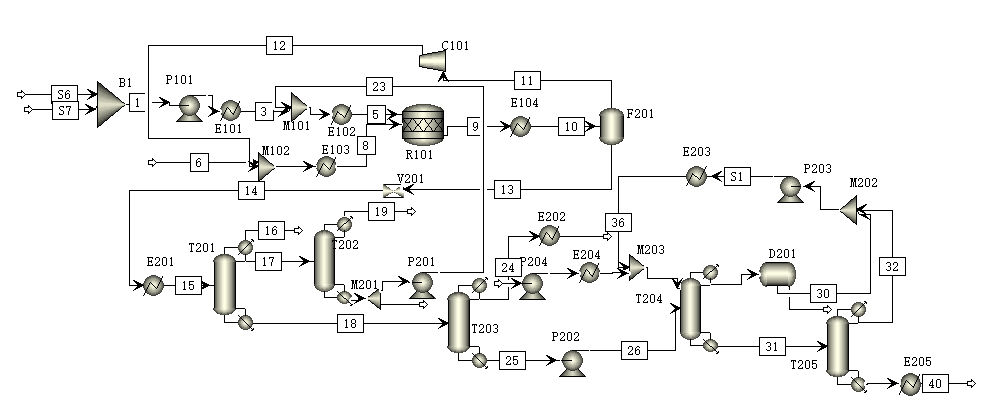
Figure S1. CHDO Scheme

Table S1. Main Streams of CHDO Process

| **Stream Number** | **Component** | **Pressure (MPa)** | **Temperature**  **(℃)** |
| --- | --- | --- | --- |
| **1** | C_3_H_8_O_3_  H_2_O | 0.1 | 25 |
| **5** | C_3_H_8_O_3_  H_2_O | 3.5 | 230 |
| **8** | H_2_ | 3.5 | 230 |
| **10** | C_3_H_8_O_2_  H_2_O  H_2_  C_3_H_6_O_2_  C_2_H_6_O_2_  CH_3_OH  C_3_H_8_O | 3.48 | 40 |
| **11** | H_2_ | 3.41 | 40 |
| **14** | C_3_H_8_O_2_  H_2_O  C_3_H_6_O_2_  C_2_H_6_O_2_  CH_3_OH  C_3_H_8_O | 0.21 | 40.7 |
| **17** | H_2_O  CH_3_OH  C_3_H_8_O | 0.14 | 108 |
| **18** | C_3_H_8_O_2_  H_2_O  C_3_H_6_O_2_  C_2_H_6_O_2_ | 0.14 | 161.8 |
| **26** | C_3_H_8_O_2_  C_2_H_6_O_2_ | 0.15 | 194.7 |
| **31** | C_3_H_8_O_2_  Cycloheptane | 0.13 | 141 |


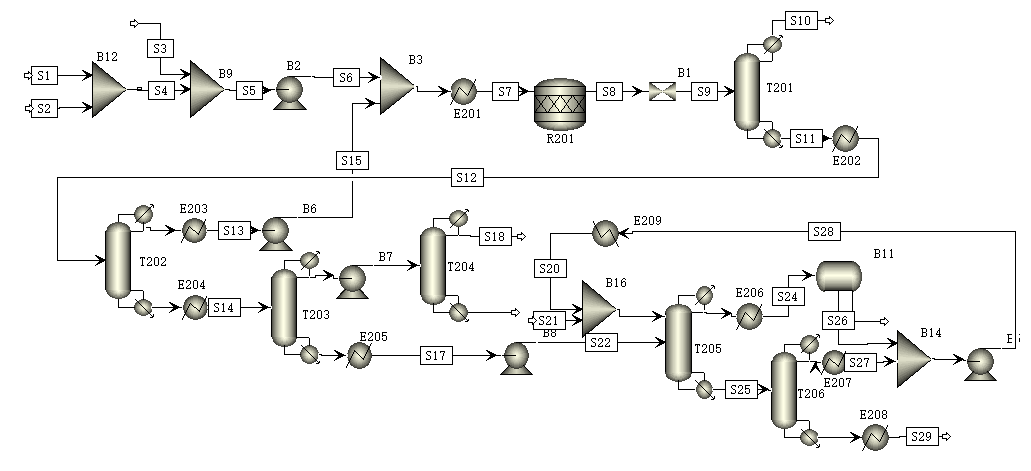


Figure S2. CTH Scheme

Table S2. Main Streams of CTH Process

| **Stream Number** | **Component** | **Pressure (MPa)** | **Temperature**  **(℃)** |
| --- | --- | --- | --- |
| **S3** | H_2_O | 0.1 | 25 |
| **S4** | C_3_H_8_O_3_  C_2_H_5_OH | 0.1 | 25 |
| **S7** | C_3_H_8_O_3_  C_2_H_5_OH  H_2_O | 3 | 200 |
| **S9** | C_3_H_8_O_2_  C_3_H_6_O_2_  C_2_H_6_O_2_  C_2_H_5_OH  CH_3_COOH  H_2_O  CH_3_OH  CH_4_  CO_2_  H_2_ | 0.4 | 133.1 |
| **S12** | C_3_H_8_O_2_  C_3_H_6_O_2_  C_2_H_6_O_2_  C_2_H_5_OH  CH_3_COOH  H_2_O | 0.15 | 118.2 |
| **S13** | C_2_H_5_OH  H_2_O | 0.21 | 40.7 |
| **S14** | C_3_H_8_O_2_  C_3_H_6_O_2_  C_2_H_6_O_2_  CH_3_COOH | 0.52 | 180 |
| **S16** | C_3_H_6_O_2_  CH_3_COOH | 0.11 | 100.7 |
| **S17** | C_3_H_8_O_2_  C_2_H_6_O_2_ | 0.12 | 192.2 |
| **S24** | C_2_H_6_O_2_  Cycloheptane | 0.11 | 40 |
| **S29** | C_3_H_8_O_2_ | 0.1 | 25 |

#### 2. Separation Sequences

Table S3. Components Information

| **Code** | **Component** | **Mole-Flow**  **/(kmol/h)** | **Boiling Point T/℃** | **The Difference of Boiling Point /℃** | **CES*** |
| --- | --- | --- | --- | --- | --- |
| **A** | H_2_ | 13.48 | -253 | 92 | 3.796 |
| **B** | CH_4_ | 2.25 | -161 | 105 | 0.728 |
| **C** | CO_2_ | 7.78 | -56 | 121 | 2.973 |
| **D** | CH_3_OH | 0.94 | 65 | 14 | 0.042 |
| **E** | C_2_H_5_OH | 24 | 79 | 21 | 1.728 |
| **F** | H_2_O | 277.61 | 100 | 18 | 0.914 |
| **G** | CH_3_COOH | 3.87 | 118 | 28 | 10.584 |
| **H** | C_3_H_6_O_2_ | 1.57 | 146 | 42 | 7.602 |
| **M** | C_3_H_8_O_2_ | 7.97 | 188 | 10 | 0.866 |
| **N** | C_2_H_6_O_2_ | 0.69 | 198 | 19 | 1.684 |

*CES: Coefficient of separation ease

According to the rules of heuristic method, giving priority to conventional distillation is the way to design the separation sequence. Because the boiling point of light components is low, pressurizing separation is applied to reduce frozen load. Separating methanol first in order to avoid the impact on the follow-up devices. Water, the maximum content of raw material, is separated preferentially to reduce the load of follow-up columns. The boiling point of propylene glycol and glycol are very close, so these two components are separated in the end. Combining the actual situation, the final separation sequence is listed below:

ABCD/EFGHMN→ EF/GHMN→GH/MN→M/N

#### 3. Equipment Selection and Design.

According to “Pressure Vessel Design Manual-4th PRESSURE VESSEL DESIGN MANUAL Fourth Edition”, the design of tanks are based on the operating conditions.

The volume of the flash tank is calculated by the formula:

$$D=0.0188{(\frac{V_{Gmax}}{u_{e}})}^{0.5}$$

Where D is the diameter of the tank, the V_Gmax_ is the maximum volume flow of the gas phase, u_e_ is the flow rate of gas in the tank.

The horizontal separator is designed by the liquid flow and standing time. The The volume of the horizontal separator is calculated by the formula:

$$D_{T}={(\frac{{2.12V}_{L}t}{C\cdot A})}^{\frac{1}{3}}$$

Where D_T_ is the diameter of the horizontal separator, C is defined as L_T_/D_T_ where L_T_ is the length of the cylindrical part, while 2.5 is the experience parameter. V_L_ is the liquid flow rate, t is the standing time, A is the variable liquid area calculated in percentage.

The design and sizing/rating of columns are calculated by Aspen Plus, including hydraulics check.

The heat exchangers are designed by calculating the heat transfer temperature difference and heat transfer area, which are defined as:

$$\Delta t_{m}=\varphi\cdot\Delta t_{R}$$

Where $\Delta t_{m}$ is the average of heat transfer temperature difference, $\varphi$ is the temperature correction factor, while $\Delta t_{R}$ is the countercurrent heat transfer temperature difference. In which way all types of current could be calculated. To improve the utilization of heat, countercurrent is used in CHDO and CTH process.

$$A=\frac{Q}{K\cdot{\Delta t}_{m}}$$

Where A is the heat transfer area, Q is the heat demand which could be calculated by specific heat capacity of each component, K is the heat transfer coefficient.

It is particularly mentioned that the margin of heat transfer area should be considered, while an experience factor is multiplied in this work.

Table S4. The amount of equipment in CHDO and CTH process

| **Device type** | **The amount of equipment** | |
| --- | --- | --- |
|  | **CHDO** | **CTH** |
| **Reactor** | 1 | 1 |
| **Column** | 5 | 6 |
| **Heat Exchanger** | 19 | 19 |
| **Pump** | 20 | 23 |
| **Flash Tank** | 1 | -- |
| **Compressor** | 1 | -- |
| **Phase Separation Tank** | 1 | 1 |

#### 4. Reactor Design

Table S5. Catalyst of CTH

| **Type** | PtFe/Y |
| --- | --- |
| **Shape** | Slice shape（Φ5×2mm） |
| **Stacking Density** | 1050kg/m^3^ |
| **Liquid Hourly Space Vel℃ity（LHSV）** | 1.5 h^-1^ |
| **Voidage** | 0.36 |

Combined with practice experience and analog computation，reactor’s dimensions are designed. According to material flow information, volume flow rate and liquid phase airspeed（LHSV）, catalyst bed volume is calculated as follows:

$$V_{C}=\frac{V_{0}}{LHSV}=\frac{3.75989}{1.5}=2.51m^{3}$$

Then we get the quality of catalyst:

$$M_{Cat}=V_{C}\times\rho_{B}=3.16\times1050=2632kg$$

In order to avoid channeling and slugging, specific design is used on tube type. The calculation model here is Homogeneous one-dimensional flat push flow model. After calculation and check computation, tube arrangement model and reactor dimension data are confirmed and listed as below.


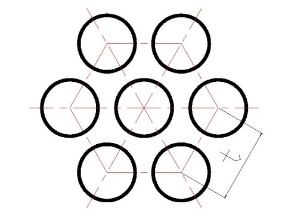


Figure S3. Tube arrangement model: Misaligned equilateral triangles

Table S6. Result of Reactor Design

| **Reactor Type** | | **Tubular Fixed Bed Reactor** |
| --- | --- | --- |
| Equipment Item Number | | R101 |
| Reaction Temperature /℃ | | 200 |
| Reaction Pressure /bar | | 30 |
| Tube pass design temperature /℃ | | 250 |
| Shell pass design temperature /℃ | | 200 |
| Tube pass working pressure /bar | | 32 |
| Shell pass working pressure /bar | | 4 |
| Reactor inner diameter /mm | | 1600 |
| Reactor tube length /mm | | 2000 |
| Reactor wall thickness /mm | | 45 |
| Catalyst Type | | PtFe/Y |
| Single Tube Hourly Space Velocity / h^-1^ | | 1.5 |
| Catalyst loading level /kg | | 2632 |
| Reaction Tube | Inner Diameter /mm | 42 |
|  | Thickness /mm | 2 |
|  | Length /mm | 1800 |
|  | Number of tubes | 667 |
|  | Arrangement Mode | Regular triangle |

Table S7. Reactor material flow parameters

| **Parameter**  **Material Flow** | **Temperature** | **Pressure** | **Density**  **ρ** | **Constant-pressure Specific Heat Cp** | **Thermal Conductivity**  **λ** | **Viscosity**  **μ** |
| --- | --- | --- | --- | --- | --- | --- |
|  | ℃ | bar | kg/m^3^ | kJ/(kg·K) | W/(m·K) | ×10^-4^Pa·s |
| **Reactants Feed** | 200 | 30 | 893.6 | 6.214 | 0.2585 | 1.7254 |
| **Product Flow** | 200 | 30 | 68.6 | 5.249 | 0.0554 | 1.4615 |
| **Entrance Boiler Feed Water** | 110 | 3.5 | 896.8 | 4.738 | 0.6835 | 2.2866 |
| **Outlet steam** | 139 | 3.5 | 1.8 | 1.907 | 0.0280 | 0.1405 |

#### 5. Economic Analysis.

In this work, the evaluation of equipment costs combined the specialty and material consumption of each kinds of equipment. Particularly, the heat-exchange area of heat exchangers and the tray or packing of columns have to be considered. According to the design and selection of standard and non-standard equipment, the equipment costs of CHDO and CTH scheme are listed in Table S7, Table S8.

Table S8. Equipment investment of CHDO scheme

| **Device Type** | **Equipment** | **Investment/**  **Thousand ＄** |
| --- | --- | --- |
| **Reactor** | R101 Hydrogenation Reactor | 144.2 |
| **Column** | T201 Liquid Phase Separation Column | 99.4 |
|  | T202 Dehydrating Column | 184.1 |
|  | T203 Acetol Separation Column | 55.6 |
|  | T204 Azeotropic Column | 60 |
|  | T205 Product Rectifying Column | 19.8 |
| **Tank** | V101 Flash Tank | 13.2 |
|  | V201 Split Phase Tank | 12.1 |
| **Compressor** | C101 Recycle Compressor | 6.1 |
| **Heat Exchanger** | E101—E104，  E201—E205  Heater and Cooler | 150.8 |
| **Pump** | P101—P107  P201—P213  Delivery Pump | 26.3 |
| **Total amount** |  | 771.6 |

Table S9. Equipment investment of CTH scheme

| **Device Type** | **Equipment** | **Investment/**  **Thousand ＄** |
| --- | --- | --- |
| **Reactor** | R101 Hydrogenation Reactor | 95.5 |
| **Column** | T201 Light Component Separation Column | 71.7 |
|  | T202 Alcohol-Water Circular Column | 259.2 |
|  | T203 Main and By-product Rectifying Column | 18.0 |
|  | T204 Acetol Rectifying Column | 7.3 |
|  | T205 Azeotropic Distillation Column | 100.4 |
|  | T206 Propylene Glycol Rectifying Column | 6.8 |
| **Tank** | V201 Split Phase Tank | 11.9 |
| **Heat Exchanger** | E101—E104，  E201—E205  Heater and Cooler | 121.7 |
| **Pump** | P101—P108  P201—P215  Delivery Pump | 30.1 |
| **Total amount** |  | 722.6 |

Table S10. Prices for chemical components

| **Chemicals** | **purity** | **Price** | **Reference** |
| --- | --- | --- | --- |
| PG | 99.8% | 2075 **$**/t | Dow Chemical official website |
| Acetic acid | 99.8% | 462 **$**/t | Run Hui chemicals co. ltd |
| Acetol | 99.1% | 46486 **$**/t | Merck Life Sciences China official website |
| EG | 99.9% | 496 **$**/t | De Ying chemicals co. ltd |
| Alcohol | 95 % | 860 **$**/t | Merck Life Sciences China official website |
| H_2_ | \ | 8 **$**/m^3^ | Merck Life Sciences China official website |

Table S11. Cost comparison of the two catalysts

| **Catalyst** | **Price** | **Annual consumption** | **Annual cost** |
| --- | --- | --- | --- |
| Ni/Cu/TiO_2_ | **$30,769**/t | **34 t** | **$1,046,146** |
| PtFe-based catalyst | **$556,923**/t | **2.2 t** | **$1,225,230** |

#### 6. Azeotropic Distillation System Design

Separation process design is one of the most prominent parts of chemical process design. The main separation equipment in this chemical process is rectifying column. According to the separation sequence analysis, there are 6 columns in the whole system to separate main and by products. After reacting in the reactor, material flow enters into Light Component Separation Column(T201). Light components such as methane are desorbed while liquid phase flows to Alcohol-Water Circular Column (T202). After distillation separation, water and alcohol are put into circulating reflux so that material is used sufficiently. Acetol and acetic acid are distilled form the top of Main and By-product Rectifying Column (T203) and separated in Acetol Rectifying Column (T204). The rest parts in Main and By-product Rectifying Column (T203) are glycol and 1,2- propylene glycol.

Because the boiling point of 1,2- propylene glycol is close to glycol. It’s really hard to separate them in conventional distillation method, in which way much more column trays need to be built, at the same time the column would be too high to set up.

To separate 1,2-propylene glycol and glycol and save energy consumptions simultaneously, the azeotropic distillation is designed including two columns and a tank. Based on analog computation results and material database of Aspen Plus, cycloheptane is chosen to be the azeotropic agent in this system.

With this Azeotropic Distillation, main product could be separated to an ideal purity, while the azeotropic agent could be separated and put into circulating reflux. After being mixed with new azeotropic agent, this part of azeotropic agent could be recycled. The Azeotropic Distillation System design is given below.


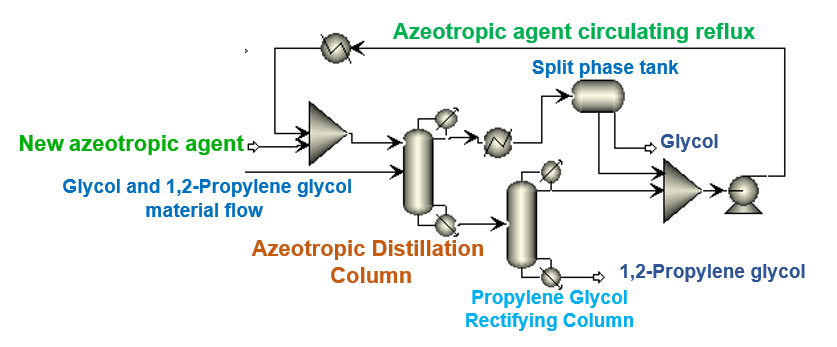


Figure S4. Azeotropic Distillation System

The azeotropic agent flow consists of Newly added cycloheptane and recycled cycloheptane. They enter into Azeotropic Distillation Column(T205) while glycol and 1,2- propylene glycol enter T205. In this step, glycol and cycloheptane are forming azeotrope and being distilled out. Then, glycol and cycloheptane would be separated in Split Phase Tank. The rest part of the cycloheptane enters into Propylene Glycol Rectifying Column(T206) with 1,2- propylene glycol. These two components would be separated in Glycol Rectifying Column(T206), which is the end of the whole process.

#### 7. Heat transfer network optimization

In this work, heat transfer network is designed to decrease energy consumption and make the maximum utilization of heat. This process based on Pinch Technology is simulated and calculated by Aspen Energy Analyzer, which matches heat flows and cold flows to decrease the use of utility. The heat transfer scheme with the lowest operating cost is optimized


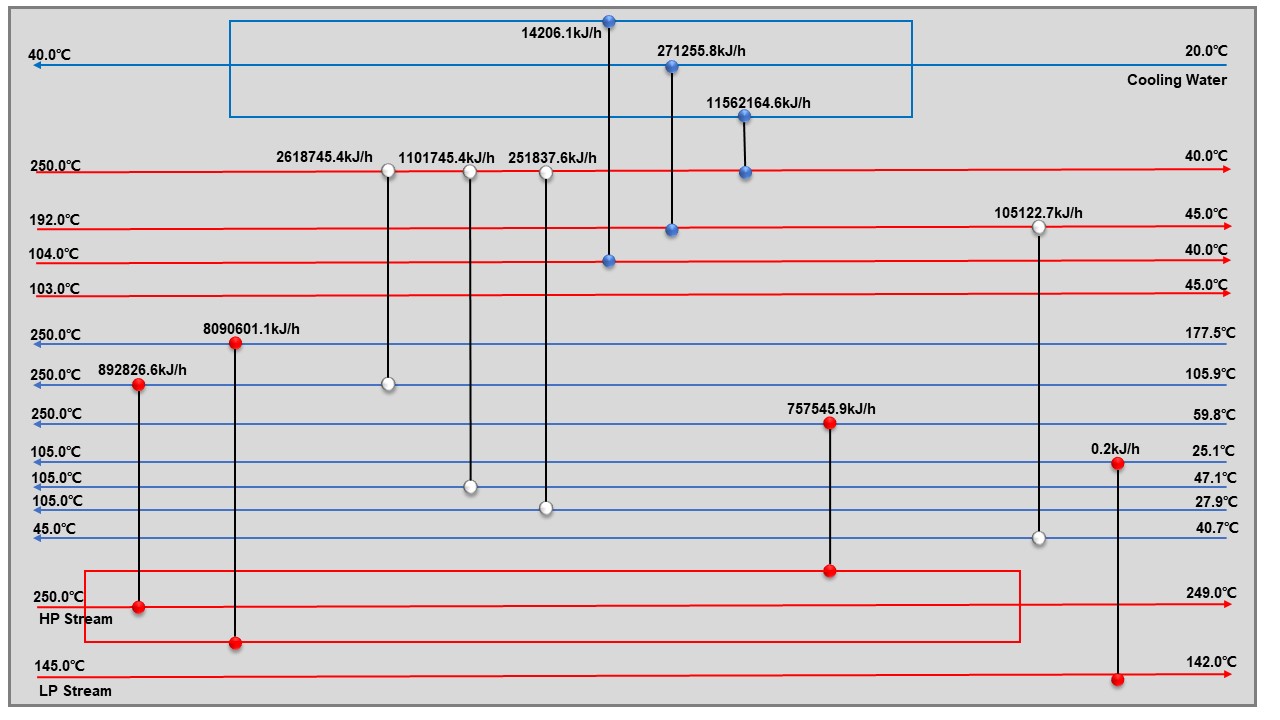


Figure S5. Heat Transfer Network for CHDO process


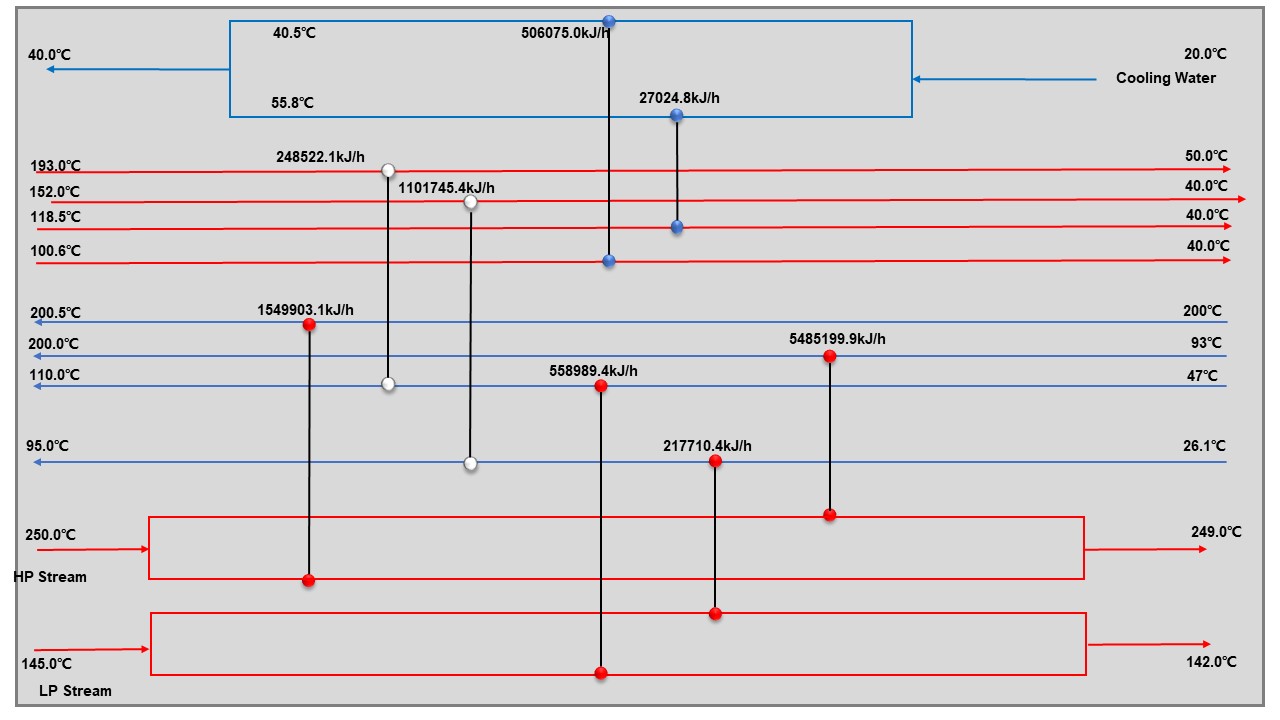


Figure S6. Heat Transfer Network for CTH process

#### 8. Reaction schemes for CHDO and CTH

For CHDO process, the transformation of glycerol usually involves the following steps. Activation of glycerol molecules is required for C-O bond cleavage. In the meantime, activation molecular H_2_ occurs on the surface of catalysts. It is generally accepted that, H atoms are then generated and spillovered on the adjacent sites of catalysts. The active H species participate in hydrogenolysis (C-O bond cleavage) for glycerol transformation.

For CTH process, different reactions are involved. Since there is no externally H_2_ available for spillover, a bifunctional catalyst material is needed for H_2_ generation from H-donors and hydrogenolysis for C-O bond cleavage.

Table S12. Reactions of CTH scheme

| **Main Reaction** | [**Selectivity**](javascript:;) |
| --- | --- |
| (1) C_2_H_5_OH+H_2_O=CH_3_COOH+2H_2_  (2) C_3_H_8_O_3_=C_3_H_6_O_2_+H_2_O  (3) C_3_H_8_O_3_+H_2_=C_3_H_8_O_2_+H_2_O  (4) C_3_H_8_O_3_+H_2_=C_2_H_6_O_2_+CH_4_O | 13.9%  11.6%  58.7%  5.1% |
| (5) C_3_H_8_O_3_+3H_2_O=3CO_2_+7H_2_ | 4.1% |
| (6) CH_4_O+H_2_=CH_4_+H_2_O | 3.6% |
| (7) C_3_H_8_O_3_+5H_2_=3CH_4_+3H_2_O | 1.9% |
